# Effects of the Presence of Adjacent Tooth and Material Type on the Marginal and Internal Adaptation of Endocrowns Fabricated by the Digital Impression Technique

**DOI:** 10.1002/cre2.70077

**Published:** 2025-02-04

**Authors:** Mehran Falahchai, Fatemeh Razavi Ardekani, Naghme Musapoor, Yasamin Babaee Hemmati, Hamid Neshandar Asli

**Affiliations:** ^1^ Dental Sciences Research Center, Department of Prosthodontics, School of Dentistry Guilan University of Medical Sciences Rasht Iran; ^2^ Department of Prosthodontics, School of Dentistry Yasuj University of Medical Sciences Yasuj Iran; ^3^ Dental Sciences Research Center, Department of Prosthodontics, School of Dentistry Qazvin University of Medical Sciences Qazvin Iran; ^4^ Dental Sciences Research Center, Department of Orthodontics, School of Dentistry Guilan University of Medical Sciences Rasht Iran

**Keywords:** CAD‐CAM, dental internal fit, dental marginal adaptation, dental material

## Abstract

**Purpose:**

To assess the effects of the presence of adjacent tooth and material type on the marginal and internal adaptation of endocrowns fabricated by the digital impression technique.

**Material and Methods:**

An endodontically treated molar tooth was used for the fabrication of endocrowns in this in vitro study. Five groups of specimens (*n* = 17) were evaluated based on the material type and presence of adjacent tooth: three groups of monolithic zirconia (Zir), lithium disilicate (LDS), and zirconia‐reinforced lithium silicate (ZLS) in the presence of adjacent teeth, and two groups of zirconia endocrowns, one in the absence of an adjacent anterior tooth (second premolar; Zir‐no ant.) and one in the absence of a posterior adjacent tooth (second molar; Zir‐no post.). Marginal and internal adaptation of endocrowns was evaluated by the silicone replica technique. Data were analyzed by ANOVA with Tukey test for pairwise comparisons and generalized estimating equation with Bonferroni correction for pairwise comparisons (α = 0.05).

**Results:**

The largest marginal gap was found in the ZLS, followed by the LDS and Zir groups (*p* < 0.05). The Zir group showed significantly higher internal adaptation than the ZLS group (*p* < 0.05); LDS had no significant difference with the ZLS and Zir groups (*p* > 0.05). The Zir group showed larger marginal and internal gaps than the Zir‐no ant. and Zir‐no post. groups (*p* < 0.05); the latter two groups had no significant difference (*p* > 0.05). All groups showed the largest gap at the pulpal and the smallest gap at the marginal and cervical areas (*p* < 0.05).

**Conclusion:**

Zirconia endocrowns showed the highest marginal and internal adaptation. Digital impression technique in absence of anterior or posterior adjacent tooth would result in higher marginal and internal adaptation of endocrowns.

## 1 Introduction

Attempts to minimize the removal of sound tooth structure in the reconstruction of endodontically treated teeth led to the introduction of endocrowns as a conservative approach for restorations of endodontically treated teeth that have lost a large portion of their structure (Al‐Dabbagh 2021). Endocrowns are mono‐block all‐ceramic coronal restorations supported by the pulp chamber and cavity margins (Papalexopoulos, Samartzi, and Sarafianou 2021; Falahchai et al. 2021). The micromechanical retention of endocrowns is obtained from the axial walls of the pulp chamber while their micro‐retention is provided by the adhesive cement (Papalexopoulos, Samartzi, and Sarafianou 2021). Evidence shows no significant difference in fracture resistance of teeth restored with endocrowns and post‐and‐core restorations (Salameh et al. 2006; Sedrez‐Porto et al. 2016). Also, in severely compromised molars, endocrowns have shown a similar or superior performance compared with direct restorations, intra‐canal posts, and conventional restorations (Salameh et al. 2006; Sedrez‐Porto et al. 2016; Mezied et al. 2022). Based on Zou, Bai, and Xiang (2018), study endocrown is mainly indicated for molars. A larger pulp chamber provides a wider bonding surface for endocrowns in molars in comparison to premolar and anterior teeth (Do et al. 2024). According to previous clinical studies (Fages et al. 2017; Otto and Mörmann 2015; Botto, Barón, and Borgia 2016; Belleflamme et al. 2017; Thomas et al. 2020), the success rate of endocrowns in molars was reported to be 72.73%–99.57% at 3‐ to 19‐year follow‐ups, also, in the survey by Topkara and Keleş (2022), mandibular molar endocrowns showed higher internal adaptation than maxillary molars, which was attributed to the morphological characteristics of the pulp chamber. Therefore, endocrowns can be considered a suitable alternative treatment option for root canal‐treated mandibular molars (Mezied et al. 2022). The computer‐aided design/computer‐aided manufacturing (CAD‐CAM) technology enables the application of new restorative materials for easier and faster fabrication of endocrowns, compared with conventional techniques (Zheng et al. 2021). Also, it has been claimed that CAD‐CAM fabrication of endocrowns enables achieving higher mechanical and optical properties (Zheng et al. 2021). Lithium disilicate glass‐ceramic is among the best materials currently available for the fabrication of ceramic restorations due to its optimal mechanical and esthetic properties (Athab Hasan and Mohammed‐Hussain Abdul‐Ameer 2023). The 10‐year survival rate of lithium disilicate endocrowns is reported to be as high as 99% (Qamar et al. 2023). ZLS glass ceramics were introduced to compensate mechanical properties of lithium disilicate in monolithic restorations (Gunal and Ulusoy 2018; Manziuc et al. 2023; Sen and Us 2018). In the composition of this glass ceramics, the lithium metasilicate (Li_2_SiO_3_) matrix is reinforced with 8% to 12% zirconia dioxide (ZrO_2_) grains, which creates a microstructure (Li_2_O‐ZrO_2_‐SiO_2_) after crystallization (Manziuc et al. 2023). This arrangement benefited from a combination of the optical properties of lithium disilicate and the mechanical properties and high polish ability of zirconia in monolithic restorations (Manziuc et al. 2023). For ZLS, partial coverage restorations success rate of 98% at 3 years was reported (Rinke et al. 2020). Also, in the study of Rinke et al., a survival rate of 69% was reported for molar restorations in a 5‐year follow‐up (Rinke et al. 2022). Zirconia is a polycrystalline ceramic material which is an excellent option for monolithic partial coverage restorations (Amini, Zeighami, and Ghodsi 2021). High flexural strength, minimal removal of tooth structure, and less wear of the opposing teeth are among the advantages of this type of restoration (Kontonasaki et al. 2019). Adding 3% and 5% yttrium‐stabilized tetragonal zirconia (Y‐TZP) can enhance esthetic and color matching (El‐Ma'aita et al. 2022). The study of El‐Ma'aita et al. reported a survival rate of 82.4% for zirconia endocrowns in a 2‐year follow‐up (El‐Ma'aita et al. 2022). Applying CAD‐CAM and intraoral scanners can yield results comparable to or superior to the conventional impression techniques (Abdel‐Azim et al. 2015). Although the accuracy of intraoral scanners under ideal conditions has been largely confirmed, their accuracy in the presence of confounders has not been well elucidated (Keeling, Wu, and Ferrari 2017). Intraoral scanners inherently require straight‐line access to record the desired region, and the desired region would not be recorded if confounders such as local anatomy (gingival level margin), the presence of adjacent teeth, tooth morphology, or limitations in optimal positioning of the scanner shaft in the oral cavity do not allow achieving straight‐line access (Keeling, Wu, and Ferrari 2017). The presence of adjacent teeth is the most prominent factor impairing optimal scanning of the mesial and distal margins (Keeling, Wu, and Ferrari 2017; Kim et al. 2022). Factors such as close margin to the adjacent tooth, height of the adjacent tooth, emergence profile of the tooth, and contour and position of the tooth affect the ability of the scanner to achieve straight‐line access to different parts of the respective tooth; resulting, the scanner records suboptimal information, and the accuracy of recording of the margins decreases (Keeling, Wu, and Ferrari 2017; Kim et al. 2022). Several studies have evaluated the material type for the fabrication of endocrowns (Zheng et al. 2021; Amini, Zeighami, and Ghodsi 2021; Hasanzade et al. 2021; El Ghoul et al. 2020; Taha et al. 2018; Sağlam, Cengiz, and Karacaer 2021). Numerous studies have also assessed the fracture resistance and behavioral and mechanical properties of different materials for the fabrication of endocrowns (Zheng et al. 2021; Taha et al. 2018; Sağlam, Cengiz, and Karacaer 2021). The highest fracture resistance has been reported for LDS restorations, compared with other materials used for endocrown fabrication such as ZLS, polymer‐reinforced ceramic, nano‐ceramic resin, polyether ketone, and feldspathic ceramic (Taha et al. 2018; Sağlam, Cengiz, and Karacaer 2021; Gresnigt et al. 2016; Nagi, Fouda, and Bourauel 2023). A literature review (Ciobanu et al. 2023) found no significant difference in the marginal adaptation of restorations fabricated from ZLS and LDS ceramics (Taha et al. 2018; Sağlam, Cengiz, and Karacaer 2021). Nonetheless, information regarding the zirconia endocrowns is scarce (Amini, Zeighami, and Ghodsi 2021). Amini, Zeighami, and Ghodsi (2021) demonstrated higher internal adaptation of ZLS endocrowns than translucent zirconia endocrowns. However, no significant difference was found in their marginal adaptation. Studies on the effect of adjacent tooth on impression accuracy are limited in number (Keeling, Wu, and Ferrari 2017). Keeling, Wu, and Ferrari (2017) demonstrated that the presence of an adjacent tooth significantly decreased the accuracy of digital impressions. However, their study was conducted on full coverage crowns, and this effect may be different on endocrowns considering their structural differences with the conventional crowns; nonetheless, no study has addressed this topic so far. Thus, the purpose of the present study was to assess the effect of the type of endocrown material and the presence of adjacent tooth on the marginal and internal adaptation of endocrowns. The null hypothesis of the study was that material type and the presence of adjacent tooth would have no significant effect on the marginal and internal adaptation of fabricated endocrowns.

## 2 Materials and Methods

In this in vitro study, the sample size was calculated to be 17 in each group according to a study by El Ghoul et al. (2020), using the ANOVA feature of PASS 11 assuming the study power of 0.99, error of 0.05, and standard deviation of 24.7 µm for the gap size.

A permanent mandibular right first molar extracted due to periodontal problems was selected for this study. After ensuring complete root formation and absence of caries, crack lines, and anatomical defects, the tooth was cleaned in an ultrasonic bath (SUPRASSON P5 Booster ultrasonic scale, Merignac Cedex, France) and stored in 0.1% thymol solution at room temperature until the experiment (Tribst et al. 2020).

**Figure 1:**
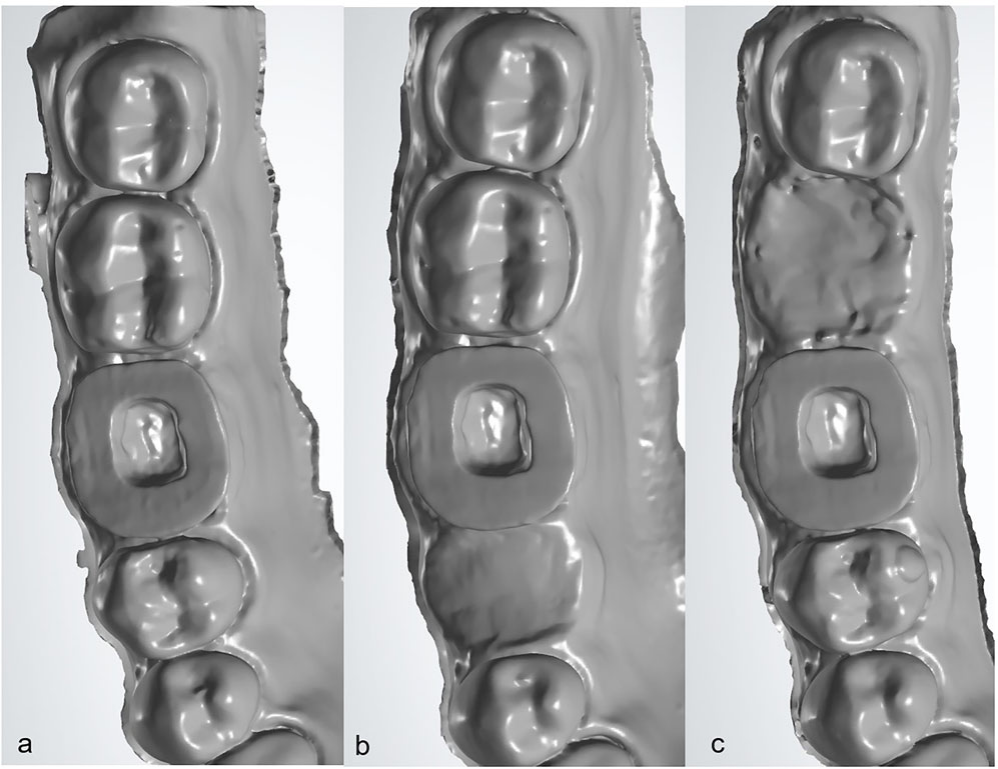
Scans of specimens (A) in the presence of adjacent teeth, (B) in the absence of adjacent anterior tooth, and (C) in the absence of posterior adjacent tooth.

For specimen preparation, the coronal part of the tooth was cut 2 mm above the cementoenamel junction, and the tooth underwent root canal therapy. The endodontically treated tooth was then mounted in a full‐arch mandibular dentiform model at the site of the first molar and fixed with utility wax (Figure 1). To prepare the tooth for an endocrown restoration, the upper part of gutta‐percha was removed 1 mm below the canal orifice by a tungsten carbide bur (Meisinger HM1; Hager & Meisinger, Düsseldorf, Germany). The occlusal surface was then prepared with a butt joint margin by using a round‐end tapered diamond bur (856; Drendel + Zweiling Diamant GmbH, Lemgo, Germany). The same bur was used to prepare the internal walls with an 8–10° taper with no undercut. The canal orifice was filled, and the pulp chamber surface was flattened with a 4 mm depth from the occlusal surface by using a flowable composite resin (Denfil Flow; Vericom, Anyang, Korea). The cavity depth was then measured by a digital caliper (Abd El‐Ghany and Sherief 2016). A round‐end red diamond bur (806314141014 fine, Jota, Ruthi, Switzerland) was used for the final finishing and rounding of all internal angles (Falahchai et al. 2021).

**Figure 2:**
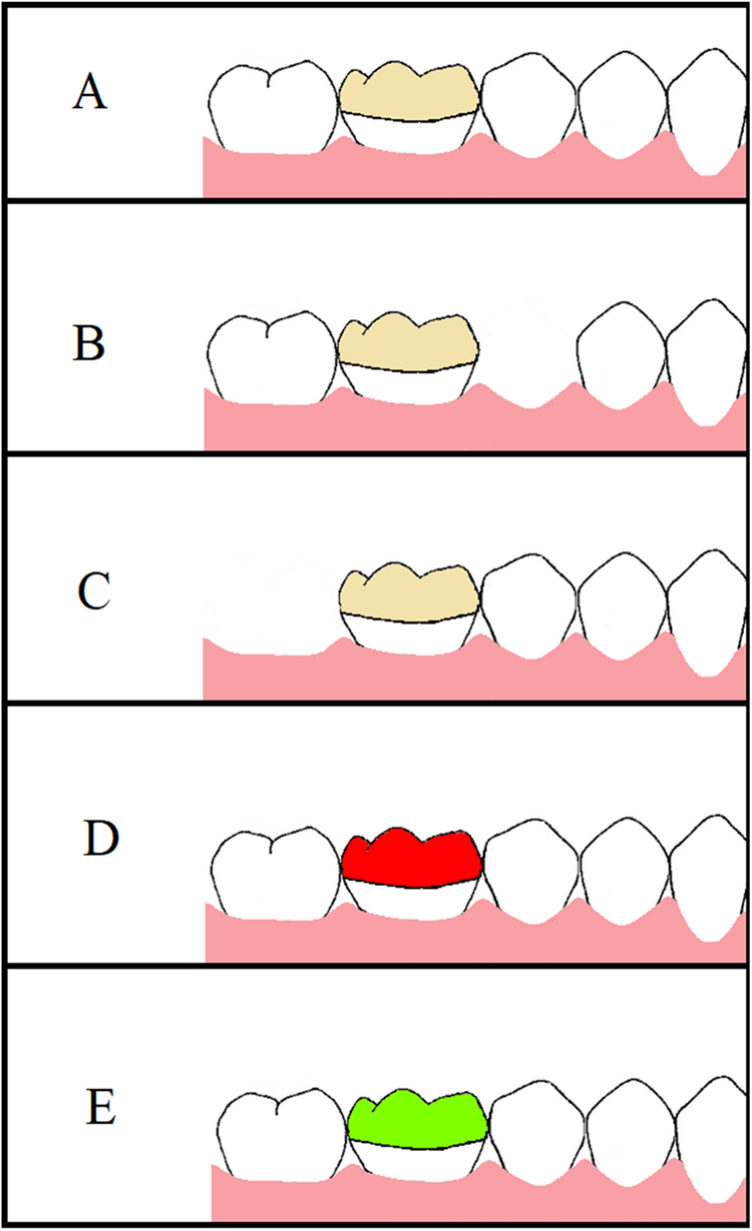
Five groups of specimens (*n* = 17). (A) monolithic zirconia in the presence of adjacent teeth, (B) monolithic zirconia‐absence of adjacent anterior tooth, (C) monolithic zirconia‐absence of the adjacent posterior tooth, (D) lithium disilicate in presence of adjacent teeth, (E) zirconia‐reinforced lithium silicate in presence of adjacent teeth.

In this study, the fabricated specimens were assigned to five groups (*n* = 17) based on the endocrown material and presence of adjacent tooth (Figure 2): (I) Zir: monolithic zirconia in the presence of adjacent teeth, (II) Zir‐no ant.: monolithic zirconia‐absence of the adjacent anterior tooth (second premolar), (III) Zir‐no post.: monolithic zirconia‐absence of adjacent posterior tooth (second molar), (IV) LDS: LDS in the presence of adjacent teeth, (V) ZLS: ZLS in the presence of adjacent teeth. In all conditions (presence of adjacent teeth, absence of adjacent anterior tooth, and absence of adjacent posterior tooth), the entire dentiform arch was digitally scanned 17 times by an intraoral scanner (Planscan; Planmeca, IL, USA) after calibration. The number of images captured was 1200–1500 per scan for standardization accuracy rate.

The scanned files were saved in STL format and transferred to Exocad software (Exocad DentalCAD, exocad GmbH, Darmstadt, Germany) for similar designing of all specimens (50 µm cement space and equal occlusogingival height) (Abd El‐Ghany and Sherief 2016). A 5‐axis milling machine (Ceramill Motion 2; Amann Girrbach, Kobach, Austria) was used for the fabrication of endocrowns. The endocrowns were fabricated by the wet grinding technique through three steps of milling. The diamond milling bur (2. 5, 1.0, and 0.6 mm), the coolant, and the lubricant were replaced after the fabrication of 10 specimens (Ahmadi et al. 2020).

**Figure 3:**
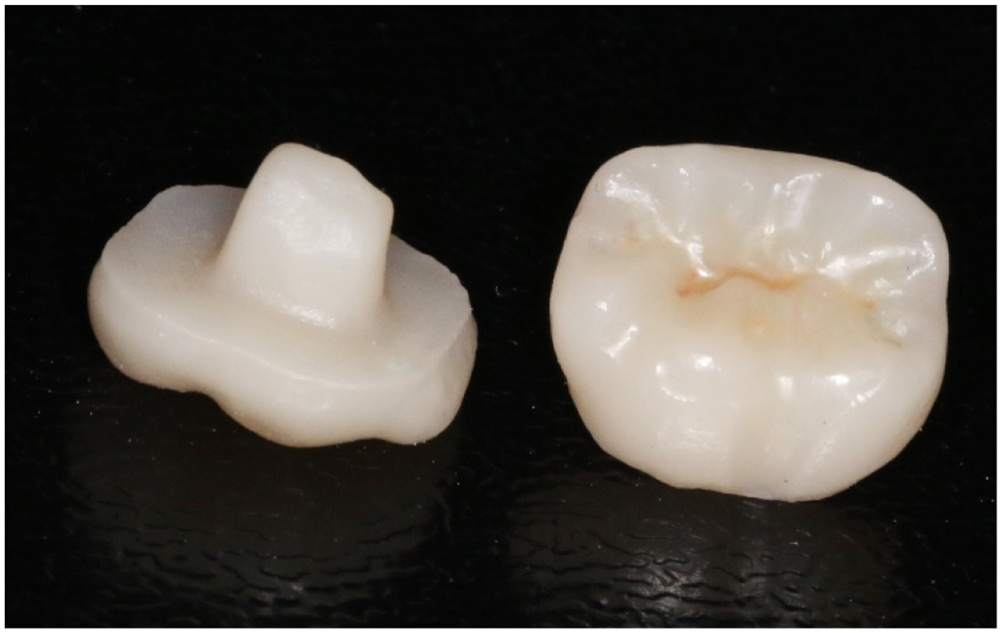
Endocrown restorations fabricated from zirconia.

In scans obtained in the presence of adjacent teeth, the endocrowns were fabricated as monolithic from three materials (*n* = 17) of zirconia (Zolid Fx multilayer; Amann Girrbach, Koblach, Austria), ZLS (Vita Suprinity; VITA Zahnfabrik, Bad Säckingen, Germany) and LDS (IPS e.max CAD; Ivoclar Vivadent, Schaan, Liechtenstein). In scans obtained in the absence of adjacent anterior/posterior tooth, the endocrowns were fabricated from monolithic zirconia (Zolid Fx multilayer; Amann Girrbach, Koblach, Austria). Endocrowns fabricated from ZLS and LDS were obtained from pre‐crystallized blanks. Crystallization was performed in a porcelain furnace (Vita Vacumat 6000 M; VITA Zahnfabrik, Bad Säckingen, Germany). ZLS blanks were initially heated to 400°C for 4 min, then heated at a heating rate of 55°C/s − 1 for 8 min until the temperature reached 840°C (Al‐Dabbagh 2021). Then, they were cooled for 8 min to 680°C with the furnace chamber closed and further cooled to room temperature. LD blanks heated at 840°C for approximately 25 min, as mentioned in the manufacturer's instructions (Papalexopoulos, Samartzi, and Sarafianou 2021; Falahchai et al. 2021). Zirconia endocrowns were fabricated from non‐sintered zirconia blocks and were subsequently sintered with the following furnace and sintering program: first ramp up: 20°C/min, temperature: 900°C, second ramp up: 10°C/min, temperature: 1450°C, hold: 120 min, first ramp down: 20°C/min, temperature: 200°C (Al‐Zordk and Saker 2020) (Figure 3). Endocrowns fabricated from ZLS and LDS were obtained from pre‐crystallized blanks. Crystallization was performed in a porcelain furnace (Vita Vacumat 6000 M; VITA Zahnfabrik, Bad Säckingen, Germany). ZLS blanks were initially heated to 400°C for 4 min, then heated at a heating rate of 55°C/s − 1 for 8 min until the temperature reached 840°C (Alao and Danish Bujang 2021). Then, they were cooled for 8 min to 680°C with the furnace chamber closed and further cooled to room temperature (Alao and Danish Bujang 2021). LDS blanks were heated at 840°C for approximately 25 min, as mentioned in the manufacturer's instructions (Hasanzade et al. 2021; Kim, Oh, and Uhm 2016).

The seating of each fabricated endocrown on the tooth was evaluated by using a dental explorer and pressure indicator paste (Fit Checker; GC, Tokyo, Japan). None of the restorations required adjustment for correct seating.

**Figure 4:**
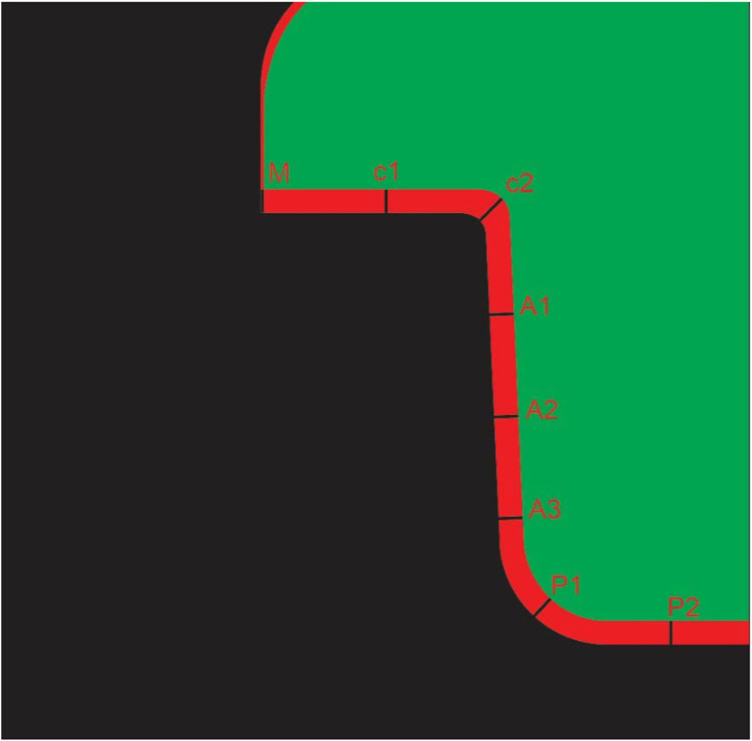
Schematic view of the points of measurement of marginal and internal gaps in the replica cross‐section; M: Marginal gap; C1 and C2: Cervical gap; A1, A2, and A3: Axial gap, P1, and P2: Pulpal gap.

Internal and marginal adaptation was assessed using the silicone replica technique. For this purpose, light‐body silicone impression material (Panasil, Kettenbach, Eschenburg, Germany) was injected into each restoration and the pulp chamber with 50 N force for 5 min. After restoration removal from the tooth, medium‐body impression material was injected over the residual light‐body material on the tooth surface for further support. Next, each silicone replica was sectioned mesiodistally and then buccolingually by a sharp scalpel. One slice with parallel walls was obtained from each of the buccal, lingual, mesial, and distal surfaces for measurement of the gap with a vertical viewing angle. In each slice, eight points were identified: one at the margin (M), two at the cervical region (C1 at the center and C2 at the cervico‐axial angle), three points in the axial wall (A1, A2, and A3) and two points at the pulpal floor (P1 at the axiopulpal angle and P2 at the center of the pulpal region), and the magnitude of gap at these points was measured by a video measuring machine (C‐Class Vision Measurement Machine; Easson optoelectronica technology, Suzhou, China) at ×132.8 magnification (Figure 4).

To assess the marginal gap, the absolute marginal gap was measured as the distance between the outermost point of the restoration margin and the external marginal line of the prepared tooth (Holmes et al. 1989). To assess the internal gap, the vertical distance between the internal crown surface and the external surface of the prepared tooth was measured, except for C2 and P1, which were the bisectors of the angle formed between the cervical region and axial wall, and the angle formed between the pulpal floor and axial wall, respectively. The measurements were repeated three times in each of the eight identified points and the average values were reported for each point. The results regarding the magnitude of the gap were reported separately for the gingival, cervical, axial, pulpal, and internal (mean of cervical, axial, and pulpal) areas and separately for each surface (buccal, lingual, mesial, and distal) in µm. All measurements were made by one examiner.

The normal distribution of data was evaluated by the Shapiro–Wilk test and the homogeneity of variances was analyzed by the Levene test. Since both the assumptions were met, data were analyzed by ANOVA followed by pairwise comparisons with the Tukey test, and also the generalized estimating equation followed by pairwise comparisons with Bonferroni correction. All statistical analyses were carried out using SPSS version 26 (INM SPSS Statistics V26, IBM Corp., NY, USA) at a 0.05 level of significance.

## 3 Results

**Figure 5:**
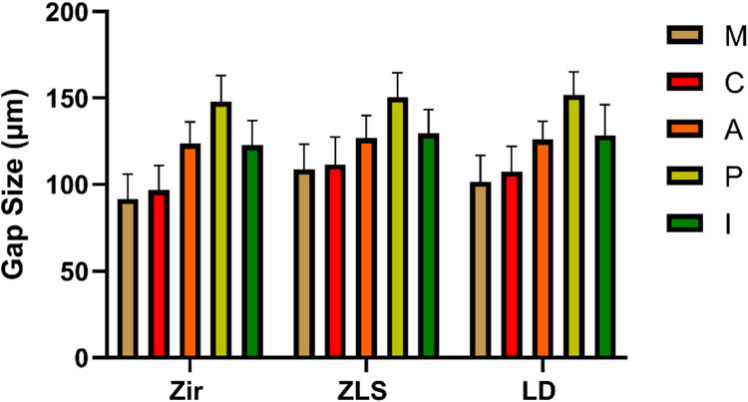
Mean gap size (in µm) at the marginal (M), cervical (C), axial (A), pulpal (P), and internal (I) areas based on the endocrown material (Zir: Zirconia, LD: IPS e.max CAD, ZLS: Vita Suprinity) (*n* = 17).

The results regarding the effect of endocrown material (Zir, ZLS, LDS) on gap size (µm) at each of the marginal (M), cervical (C), axial (A), pulpal (P), and internal (I) areas (Figure 5) by ANOVA revealed that the effect of material type was significant on M, C, and I gaps. Pairwise comparisons by the Tukey test revealed that at the M area, the smallest gap was recorded in Zir, followed by LDS, and then ZLS (*p* < 0.05). At C, Zir showed the smallest gap (*p* < 0.01). Also, Zir showed a smaller internal gap than ZLS (*p* < 0.05). No significant difference was noted when comparing other groups (*p* > 0.05). Table 1 presents the results regarding the comparison of the groups at each of the M, C, A, P, and I areas separately in the mesial and distal surfaces.

**Table 1:** Mean and standard deviation of gap size (in µm) and its comparison at the marginal (M), cervical (C), axial (A), pulpal (P), and internal (I) areas based on the endocrown material and presence of adjacent tooth, and comparison of marginal (M), cervical (C), axial (A), pulpal (P), and internal (I) gaps separately in the mesial (Me) and distal (Di) surfaces (*n* = 17).

Location	Region	Zir Mean ± SD	ZLS Mean ± SD	LD Mean ± SD	*p* value (F)*	Zir Mean ± SD	Zir‐No Ant Mean ± SD	Zir‐No Post Mean ± SD	*p* value (F)*
Me	M	93.08 ± 15.11^Aa^	111.01 ± 19.12^Ab^	105.02 ± 17.06^Aab^	0.013 (4.80)	93.08 ± 15.11^Aa^	69 ± 10.44^Ab^	90.02 ± 13.54^Aa^	< 0.001 (16.84)
	C	98.98 ± 18.90^Aba^	121.62 ± 14.63^ABb^	112.95 ± 1298^Ab^	< 0.001 (8.99)	98.98 ± 18.90^Aa^	78.63 ± 11.66^Ab^	91.97 ± 15.95^Aa^	0.002 (7.29)
	A	129.14 ± 11.64^Ba^	126.36 ± 14.97^Ba^	124.34 ± 9.13^Ba^	0.517 (0.67)	129.14 ± 11.64^Ba^	113.13 ± 10.12^Bb^	111.51 ± 18.81^Bb^	0.001 (8.18)
	P	151.72 ± 13.14^Ca^	152.98 ± 11.17^Ca^	155.55 ± 11.02^Ca^	0.631 (0.46)	151.72 ± 13.14^Ca^	140.88 ± 19.46^Cab^	133.02 ± 17.90^Cb^	0.009 (5.16)
	*p* value** (F)	< 0.001 (56.91)	< 0.001 (23.34)	< 0.001 (50.42)		< 0.001 (56.91)	< 0.001 (101.77)	< 0.001 (24.68)	
	I	126.43 ± 14.39^a^	133.63 ± 16.94^a^	130.80 ± 18.30^a^	0.451 (0.81)	126.43 ± 14.39^a^	110.56 ± 14.02^b^	112.19 ± 16.03^b^	0.005 (5.89)
Di	M	91 ± 16.38^Aa^	110.01 ± 11.2^Ab^	102 ± 11.38^Ab^	0.001 (8.86)	91 ± 16.38^Aa^	88 ± 12.99^Aa^	73 ± 14.16^Ab^	0.002 (7.44)
	C	99.77 ± 13.99^Aa^	100.87 ± 14.98^Aab^	111.92 ± 14.86^Ab^	0.035 (3.59)	99.77 ± 13.99^Aa^	93.65 ± 13.38^Aa^	76.08 ± 16.14^Ab^	< 0.001 (12.14)
	A	123.36 ± 13.84^Ba^	128.57 ± 14.54^Ba^	127.86 ± 10.10^Ba^	0.453 (0.81)	123.36 ± 13.84^Ba^	116.38 ± 12.06^Ba^	115.25 ± 10.28^Ba^	0.119 (2.23)
	P	148.38 ± 16.49^Ca^	151.18 ± 16.23^Ca^	150.30 ± 15.98^Ca^	0.876 (0.13)	148.38 ± 16.49^Ca^	138.29 ± 15.38^Ca^	139.15 ± 13.58^Ca^	0.111 (2.30)
	*p* value (F)**	< 0.001 (48.66)	< 0.001 (40.74)	< 0.001 (42.69)		< 0.001 (48.66)	< 0.001 (49.35)	< 0.001 (92.40)	
	I	123.96 ± 15.60^a^	126.66 ± 12.24^a^	130.06 ± 17.38^a^	0.509 (0.68)	123.96 ± 15.60^a^	116.24 ± 14.79^ab^	110.21 ± 11.75^b^	0.024 (4.04)

*Note:* SD, standard deviation; Zir, Zirconia; LD, IPS e.max CAD; ZLS, Vita Suprinity; Zir‐No Ant, Zirconia‐no anterior tooth (second premolar); Zir‐No Post, Zirconia‐no posterior tooth (second molar). Values with different uppercase letters in the same column are significantly different (*p* < 0.05). Those with different lowercase letters in the same row are significantly different (*p* < 0.05).

* ANOVA and Tukey post hoc test.

** GEE and Bonferroni correction post hoc test.

**Figure 6:**
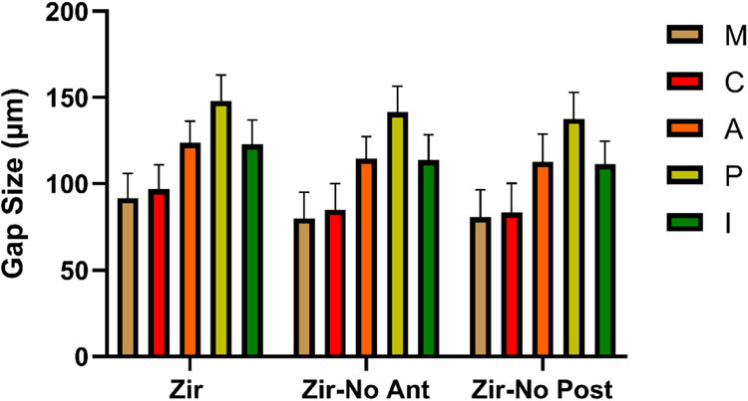
Mean gap size (in µm) at the marginal (M), cervical (C), axial (A), pulpal (P), and internal (I) areas based on the presence of adjacent tooth (Zir: Zirconia, Zir‐No Ant: Zirconia‐no anterior teeth (second premolar), Zir‐No Post: Zirconia‐no posterior teeth (second molar) (*n* = 17).

Comparison of Zir, Zir‐no ant., and Zir‐no post. groups regarding the gap size at each of the M, C, A, P, and I areas (Figure 6) by ANOVA revealed that the effect of the presence of adjacent anterior and posterior teeth was significant in all areas (*p* < 0.001). Pairwise comparisons showed that in each area, the Zir group showed significantly larger gaps than the Zir‐no ant. and Zir‐no post. groups (*p* < 0.05); however, the difference between the Zir‐no ant. and Zir‐no post. groups was not significant (*p* > 0.05). Table 1 shows the comparison of the groups at each of the M, C, A, P, and I areas separately in the mesial and distal surfaces.

Comparison of the mean gap at M, C, A, and P areas in each study group (Zir‐no post., Zir‐no ant., Zir, ZLS, and LDS) by ANOVA revealed significant differences in all groups among different areas (*p* < 0.001). Pairwise comparisons by the Tukey test indicated the largest gap at P and the smallest gap at M and C (*p* < 0.001). The difference between M and C was not significant (*p* > 0.05). Table 1 presents the results of the comparison of areas separately in the mesial and distal surfaces.

Comparison of gap size among the buccal, lingual, mesial, and distal surfaces separately at each of the M, C, A, P, and I areas in each study group by ANOVA indicated significant differences at M between Zir‐No ant. and Zir‐no post., groups and at C among Zir‐no ant., Zir‐no post., ZLS, and LDS groups (*p* < 0.05). Pairwise comparisons by the Tukey test revealed greater gaps at the mesial than the distal surface at the M and C areas in the Zir‐no post group and at the C area in the ZLS group. Also, the mesial surface showed a smaller gap than the distal surface at the C area and a smaller gap than the distal and buccal surfaces at the M area in the Zir‐no ant. group (*p* < 0.05). In the LDS group, the buccal surface indicated a smaller gap than the mesial and distal surfaces at the C area (*p* < 0.05). The difference among surfaces was not significant in other areas (*p* > 0.05).

## 4 Discussion

The results refuted the null hypothesis of the study regarding the insignificant effect of material type and the presence of adjacent teeth on the marginal and internal adaptation of endocrowns. In the present study, one extracted natural tooth was used for the fabrication of all specimens for standardization. Therefore, the effects of confounding factors such as differences in the natural anatomy of the teeth and different preparation designs on the results were eliminated. The use of natural teeth, instead of artificial teeth, enables better simulation of the clinical setting such as the presence of enamel and dentin, as well as the natural contour and structure of the pulp chamber and canal shape (Taha et al. 2018). On the other hand, artificial specimens have different structures and surface properties compared with natural teeth, and therefore have different optical responses which can affect the digital scanning process (Ferrini et al. 2019).

According to previous studies (Güth et al. 2013; Güntekin et al. 2024; Chiu et al. 2020), by increasing the number of images in intraoral scanning, the details of an arch will be recorded with higher accuracy. However, exceeding 1600 images/scan can lead to a software error (Gómez‐Polo et al. 2023). The highest accuracy rate was calculated at 1200–1500 images per scan based on the study of Güntekin et al. (2024). Therefore, in this study, the number of images captured was 1200–1500 per scan for standardization of accuracy rate.

The silicone replica technique was used for the assessment of internal and marginal adaptation, which has been extensively used for this purpose in the literature as a reliable technique (Boitelle et al. 2014; Park et al. 2017; Nawafleh et al. 2013; Licurci et al. 2022; Di Fiore et al. 2023). It is simple, low‐cost, and reproducible (Di Fiore et al. 2023). It allows the assessment of adaptation at several internal and marginal points. Also, its accuracy can be maximized by increasing the number of assessment points (Di Fiore et al. 2023). Furthermore, it is noninvasive and non‐destructive, and therefore allows repeated assessments and evaluation of clinical specimens and comparison with in vitro findings. In the current study, eight points of each slice (a total of 32 points) were assessed under a video measuring machine, which provides high‐resolution images without contacting the specimen for highly accurate dimensional measurements (Wu et al. 2021).

The magnitude of marginal gaps ranged from 69 ± 10.44 µm in the Zir‐no ant group at the mesial aspect to 111.01 ± 19.12 in the ZLS group at the mesial aspect. The mean internal gap ranged from 110.21 ± 11.75 in the Zir‐no post group at the distal aspect to 133.63 ± 16.94 in the ZLS group at the mesial aspect. According to previous studies, a mean internal gap size between 70 and 120 µm and a marginal gap size smaller than 120 µm are clinically acceptable (Abdel‐Azim et al. 2015; Măroiu et al. 2021; Arezoobakhsh et al. 2020; Tidehag, Ottosson, and Sjögren 2014). Thus, it may be concluded that the marginal gap size in all groups and internal gap size in Zir‐no ant and Zir‐no post were within the clinically acceptable range; in other groups, the internal gap was more than the clinically acceptable range, which can be related to the absence of a standard protocol for the fabrication process and differences in scanner type, milling machine, cement space, and technique of measurement of discrepancy (Abd El‐Ghany and Sherief 2016).

Several factors such as restoration material and its mechanical properties, the technique of assessment of the gap, and the assessed points can affect the measured gap size (Nawafleh et al. 2013; Di Fiore et al. 2023; Neppelenbroek 2015). Assessment of the effect of endocrown material on the mean marginal and internal gaps in the current study revealed that the marginal gap was the highest in the ZLS group, followed by the LDS and Zir groups. In general, zirconia showed smaller gaps than the ZLS. Unlike the present study, Amini, Zeighami, and Ghodsi (2021) compared the marginal and internal gaps of endocrowns fabricated from translucent zirconia and ZLS and found that the marginal gap was significantly smaller in the ZLS group; however, the difference in the internal gap was not significant between the two materials. Previous studies found no significant difference between ZLS and LDS endocrowns in terms of marginal (Hasanzade et al. 2021; Taha et al. 2018; Sağlam, Cengiz, and Karacaer 2021) and internal (Hasanzade et al. 2021) adaptation. Such conflicting results can be due to differences in the type of zirconia (translucent in the study by Amini, Zeighami, and Ghodsi ( 2021), the brand of ZLS in the study by Taha et al. (2018) (Celtra‐Duo), and areas and technique of measurement of the mean gap (Amini, Zeighami, and Ghodsi 2021; Hasanzade et al. 2021; Sağlam, Cengiz, and Karacaer 2021). The smaller gap size in zirconia restorations of the present study may be attributed to the higher flexural strength of monolithic zirconia compared with LDS and ZLS (Abd El‐Ghany and Sherief 2016; Almohammed, Alshorman, and Abu‐Naba'a 2023). Higher flexural strength is associated with greater resistance to chipping and crack formation in the fabrication process (Goujat et al. 2018). Also, a reduction in mechanical properties such as modulus of elasticity and hardness of restoration would result in greater bur penetration during preparation and subsequently greater removal of material and compromise the adaptation of final restoration as such (Coldea, Swain, and Thiel 2013). Moreover, previous studies (Taha et al. 2018; Sağlam, Cengiz, and Karacaer 2021) reported higher fracture resistance and mechanical properties for LDS compared with ZLS endocrowns, which explains the higher adaptation of LDS restorations.

The present results indicated that the gap size of zirconia endocrowns in the presence of adjacent teeth was significantly higher than that of endocrowns scanned in the absence of adjacent anterior/posterior teeth. However, the difference in the marginal and internal gap was not significant when comparing the absence of anterior versus posterior tooth. Kim et al. (2022) evaluated the effect of the presence of an adjacent tooth on the accuracy of impression‐making by an intraoral scanner for the fabrication of inlay restorations. The results showed an increase in impression accuracy in the absence of an adjacent tooth. Huang, Son, and Lee (2021) assessed the effect of distance between the abutment tooth and the adjacent tooth on the accuracy of intraoral scanning and found that increasing the distance by more than 1.5 mm increased the scanning accuracy. Nonetheless, to the best of the authors' knowledge, no previous study assessed the effect of the presence of adjacent teeth on the marginal and internal gaps of endocrowns. The present results revealed a smaller marginal gap at the mesial than the distal surface in the Zir‐no ant. group and a smaller marginal gap at the distal than the mesial surface in the Zir‐no post. group. The smaller marginal gap at the distal surface in the absence of an adjacent posterior tooth and the smaller marginal gap at the mesial surface in the absence of an adjacent anterior tooth may be explained by greater freedom of positioning of the scanner shaft. Accordingly, the sharpness and accuracy of the scans can be maximized by precisely adjusting the position of the scanner (Keeling, Wu, and Ferrari 2017).

A comparison of the mean gap at different areas within each group indicated the largest gap at the pulpal wall, which was in line with previous findings (Amini, Zeighami, and Ghodsi 2021; Shin et al. 2017; Hasanzade et al. 2021; Abd El‐Ghany and Sherief 2016). The increased gap size at the pulpal wall can be attributed to the increased depth of the scan in this area. The distance between the scanner head and the pulpal area is greater than the distance in other areas. Considering the limitations in scanning depth, the obtained images from this scan may be blurred, which would decrease the adaptation and increase the gap size (Amini, Zeighami, and Ghodsi 2021). Moreover, insignificant taper during preparation may further complicate the precise scanning of the pulpal area (El Ghoul et al. 2020).

Unlike conventional impression technique where a light‐body impression material can accurately detach soft tissue and capture deep areas, one of the frequent problems with digital impression technique is that it is difficult to accurately detect the entire margin in deep areas (Zimmermann et al. 2015; Imburgia et al. 2017; Aragón et al. 2016; Lawson and Burgess 2015; Mangano et al. 2017). Moreover, previous studies (Ardekani et al. 2024; Shin et al. 2017; Gurpinar and Tak 2022; Gaintantzopoulou and El‐Damanhoury 2016) have suggested that increasing the depth of the cavity compromises the marginal and internal adaptation in endocrowns fabricated with optical scanners.

This study had an in vitro design. Due to eliminating clinical intervention factors such as oral fluids, soft tissue (tongue, cheek, and gingiva), patient movements during the scanning process, and the limitation of mouth opening which is significant in the posterior mandible area, different scenarios may be encountered in the clinical setting (El Ghoul et al. 2020). Also, the accuracy of the silicone replica technique is influenced by the accuracy of the impression material used, measurement precision, and the number of assessed points. Therefore, its accuracy may be questionable compared to three‐dimensional methods that uniformly assess all internal and marginal points. Moreover, this study was conducted on mandibular molars. Its results may not apply to anterior, premolars, and maxillary posterior molars teeth due to differences in morphological and anatomical characteristics of the pulp chamber and different scanner access in each of the other areas (Do et al. 2024; Topkara and Keleş 2022). Future clinical and in vitro studies with a larger sample size on other maxillary and mandibular teeth are recommended to use different techniques for measurement of marginal and internal adaptation to obtain more accurate results.

## 5 Conclusion

The highest marginal adaptation was found in the Zir, followed by the LDS and then ZLS endocrowns. Zir restorations showed greater internal adaptation than ZLS endocrowns; while LDS restorations had no significant difference with Zir and ZLS endocrowns in this respect. In the absence of anterior or posterior teeth, greater internal and marginal adaptation was found. Marginal adaptation was higher in the surface adjacent to the edentulous area compared to the surface where the adjacent tooth was present, but the evaluation of internal adaptation showed no significant difference among different surfaces. Comparison of gap size at different areas within each group showed the largest gap at the pulpal and the smallest gap at the marginal and cervical areas. The difference in gap size between the marginal and cervical areas was not significant.

## Author Contributions

**Mehran Falahchai:** conceptualization, methodology, writing–review and editing. **Fateme Razavi Ardakani:** resources, investigation, visualization. **Naghmeh Musapoor:** methodology, visualization. **Yasamin Babaee Hemmati:** writing–original draft, data. **Hamid Neshandar Asli:** funding acquisition, project administration, supervision.

## Ethics Statement

This is an in vitro study (ethics code: IR.GUMS.REC.1399.562).

## Consent

The authors have nothing to report.

## Conflicts of Interest

The authors declare no conflicts of interest.

## Data Availability

The data sets used and/or analyzed during the current study are available from the corresponding author on reasonable request. Also, the data sets supporting the conclusions of this article are included within the article.
